# Pathogenic characterization of *Phialophora submersa*, a new black yeast isolated from freshwater sediments in Spain

**DOI:** 10.3389/fcimb.2025.1620047

**Published:** 2025-07-14

**Authors:** Ana Fernández-Bravo, Laura Camuña-Pardo, Marta Sanchis, Youssef Ahmiane, Javier Capilla, Josepa Gené

**Affiliations:** ^1^ Mycology and Environmental Microbiology Unit, Faculty of Medicine and Health Science, Rovira i Virgili University, & Pere Virgili Health Research Institute (IISPV), Reus, Spain; ^2^ Serra Húnter Fellow, Mycology and Environmental Microbiology Unit, Faculty of Medicine and Health Science, Rovira i Virgili University, & Pere Virgili Health Research Institute (IISPV), Reus, Spain

**Keywords:** *Phialophora*, environmental species, macrophages, immune response, virulence, antifungal susceptibility, stress responses

## Abstract

*Phialophora submersa* is a recently described black yeast species (*Chaetothyriales*), isolated from freshwater sediments in Catalonia (Spain). It is closely related to *P. americana* and *P. verrucosa*, two opportunistic pathogens known to cause subcutaneous infections in humans and animals. This study investigates the pathogenic potential of *P. submersa*, its *in vitro* susceptibility to clinically relevant antifungal agents, and its response to various cellular stressors. Using a murine macrophage (J774A.1) infection model, we evaluated phagocytosis, intracellular survival, cell damage, and the expression of six immune-related genes (*TNF-α*, *CCL20*, *RELA*, *TP53*, *NLRP3*, *IL-1β*), in comparison with *P. americana* and *P. verrucosa*. The results showed that *P. submersa* induced higher phagocytosis rates in murine macrophages than the *P. verrucosa*, although lower than *P. americana*. Cell damage, intracellular survival, and expression of the immune-related genes were higher after macrophage infection with *P. verrucosa* than with *P. submersa* and *P. americana*, which exhibited comparable profiles. All three species displayed similar antifungal susceptibility profiles, being susceptible to most azoles (except fluconazole), terbinafine, and echinocandins (with reduced efficacy against *P. verrucosa*), but showed moderate resistance to flucytosine, amphotericin B, and olorofim. The resistance of *P. submersa* to stress was strain-dependent, with only one strain exhibiting notable resistance to multiple stressors. This research provides new insights into the biology of *P. submersa*, including its potential as a human pathogen, and the molecular factors that could drive an infection process.

## Introduction

*Phialophora* is an ascomycetous genus belonging to the family *Herpotrichiellaceae* within the order *Chaetothyriales*. It comprises a group of melanized species characterized by brown, septate, and branched hyphae, and the production of conidia from flask-shaped or elongated conidiogenous cells with characteristic vase- to funnel-shaped darkly pigmented collarettes (De Hoog et al., 2020). Most *Phialophora* species are commonly found in the environment, where they play a role in the decomposition of plant material; however, they are also recognized for their pathogenic potential in humans (Li et al., 2017; De Hoog et al., 2020; Song et al., 2021). Among them, *P. verrucosa* and *P. americana* are well-documented causative agents of subcutaneous phaeohyphomycosis in both humans and animals. These species have also been associated, albeit less frequently, with chromoblastomycosis, a chronic fungal infection characterized by verrucous skin lesions containing brownish multiseptated fungal cells in the subcutaneous tissue (Singh et al., 1997; Borrás et al., 2022; Martini et al., 2022; Yiallouris et al., 2024). *Phialophora* infections usually occur through traumatic inoculation of fungal elements via skin, which are able to survive and proliferate into the tissue in immunocompromised, but also in apparently healthy individuals (Tong et al., 2013; De Hoog et al., 2020; Ahmed et al., 2021; Ply et al., 2023). Other opportunistic species in the genus recently associated with human disease are *P. chinensis*, *P. expanda*, *P. macrospora*, and *P. tarda* (De Hoog et al., 2020; Ahmed et al., 2021; Pruvot et al., 2022; Ply et al., 2023). Notably, multiple clinical cases involving *Phialophora* species have been linked to patients with immune system impairments, particularly those involving CARD9 (Caspase Recruitment Domain-containing protein 9) deficiencies (Liang et al., 2015; Ahmed et al., 2021; Song et al., 2021).

In addition to the clinical relevance, *Phialophora* species have been investigated for their ecological functions, particularly their capacity to degrade lignin and other complex organic compounds in their natural environments (Kwasna et al., 2021; Yasanthika et al., 2025). This dual role of *Phialophora*, as both environmental decomposers and opportunistic pathogens, makes the genus particularly intriguing from both medical mycology and environmental microbiology perspectives (Yiallouris et al., 2024). The presence of melanin in the cell wall enables *Phialophora* species to thrive in hostile environments, including low oxygen conditions and high irradiation exposure, and likely enhances their resistance to certain host immune responses (Guarro et al., 1999; Passero et al., 2021).

A recent survey exploring ascomycete diversity in freshwater sediments revealed *Phialophora* strains in both oligotrophic and eutrophic areas of several Spanish rivers, thereby broadening our understanding of the ecological niches occupied by potentially pathogenic fungi (Torres-Garcia et al., 2023). Approximately half of the isolates were identified as *P. americana*, while the remaining isolates represented a new lineage for the genus that was named *P. submersa*. Despite the genetic divergence, the two species exhibited only subtle morphological differences to be distinguished (Torres-Garcia et al., 2023). However, variations in their cardinal temperatures for growth were noted; *P. americana* displayed the optimum and maximum temperatures at 30 and 37 °C, as is typical for many other species in the genus, whereas *P. submersa* these were 25 and 30 °C, respectively (Li et al., 2017; Torres-Garcia et al., 2023). Irrespective of the lack of *in vitro* thermotolerance at 35─37 °C of *P. submersa*, but considering the clinical relevance of the *Phialophora* species, we consider interesting to investigate the pathogenic potential of *P. submersa*, particularly in comparison to the well-established human opportunists *P. verrucosa* and *P. americana*.

This study investigates the pathogenic mechanisms of the environmental species *P. submersa*, focusing on its ability to survive phagocytosis, trigger immune responses, and adapt to diverse conditions. By comparing it with *P. verrucosa* and *P. americana*, we highlight key similarities and differences in pathogenicity among *Phialophora* species. We also evaluate its susceptibility to commercial antifungal drugs and its response to stressors affecting the cell wall, membrane, and osmotic balance. These findings offer new insights into the biology of *P. submersa* and its potential role as a human pathogen.

## Materials and methods

### *Phialophora* strains

All *Phialophora* strains used in this study were isolated from freshwater sediments, as described by Torres-García et al (Torres-Garcia et al., 2023), with the exception of *P. verrucosa*, which was sourced from the fungal collection at the Westerdijk Fungal Biodiversity Institute (CBS, Utrecht, the Netherlands). The former strains have been deposited in the fungal culture collection of the Medicine Faculty at the Rovira i Virgili University (FMR, Reus, Spain), and their metadata are summarized in **Table 1**.

**Table 1:** *Phialophora* strains used in the study.

Species	Strain	Origin
*P. americana*	FMR 18552	Ter River, Sant Joan de les Abadesses, Girona, Spain
	FMR 18630	Ter River, Vilanova de Sau, Barcelona, Spain
*P. submersa*	FMR 17150^T^	Les Obagues Stream, Cornudella del Montsant, Tarragona, Spain
	FMR 18985	Llobregat River, Guardiola del Berguedà, Barcelona, Spain
	FMR 18986	Llobregat River, Guardiola del Berguedà, Barcelona, Spain
	FMR 18997	Llobregat River, Castellar de n’Hug, Barcelona, Spain
	FMR 18998	Llobregat River, Castellar de n’Hug, Barcelona, Spain
*P. verrucosa*	CBS 140325^T^	Phaeohyphomycosis leg, China

^T^, Type strain of the species.

### Cell lines and conditions

The J774.1A mouse macrophage cell line (BALB/C) was chosen for the infection experiments. Cells were cultured as adherent cells in DMEM (PAA Laboratories GmbH), supplemented with 10% fetal bovine serum (FBS, PAA Laboratories GmbH, Munich, Germany) and 1% penicillin-streptomycin (P/S, PAA Laboratories GmbH, Munich, Germany), and incubated at 37°C with 5% CO_2_. Prior to infection assays, cells were seeded in tissue culture plates using DMEM lacking FBS and P/S at a density of 0.5 × 10^6^ cells/mL, aiming to achieve a final concentration of 1 × 10^6^ cells/mL after 24 h (Fernández-Bravo and Figueras, 2022).

### Infection

The J774.1A macrophage cell line was infected with *P. americana* (FMR 18552, FMR 18630)*, P. submersa* (FMR 17150, FMR 18986), and *P. verrucosa* (CBS 140325) (**Table 1**). Fungal strains were grown overnight in DMEM without FBS and P/S, at a multiplicity of infection (MOI) of 10, representing the ratio of conidia to target cells. Cultures were incubated at 37°C in a 5% CO_2_ atmosphere and sampled at specific time points depending on the experimental conditions.

### Phagocytosis and intracellular survival

J7741.A cells were seeded in 24-well plates and infected with the different *Phialophora* strains at an MOI of 10 and were incubated for 1 h at 37°C with 5% CO_2_. After infection, macrophages were washed with PBS, and the number of colonies associated with the cells was determined by serial dilution and plating. Infected macrophages were washed to eliminate extracellular conidia. Following the wash, the number of intracellular conidia was determined. The percentage of phagocytosis was calculated based on the colony counts after 1 h post-infection in comparison with the inoculum (Fernández-Bravo et al., 2019).

For the intracellular survival assay, macrophages were infected with the strains as described, and fungal loads were measured 1 h post-infection. The remaining infected macrophages were then incubated in fresh DMEM for an additional 4 to 6 h at 37°C with 5% CO_2_. After incubation, cells were washed with PBS, and the number of intracellular conidia was counted. The percentage of fungal survival was calculated based on the number of colonies after the 4 to 6 h incubation period in comparison with 1 h post-infection (Fernández-Bravo et al., 2019).

### Cell damage assay

After infection of J7741.A cells at an MOI of 10 with the cultures, supernatants were collected at 3 and 5 h post-infection. Cell damage was assessed by quantifying the lactate dehydrogenase (LDH) enzyme released into the supernatants using the Cytox 96 Non-Radioactive Cytotoxicity Assay (Promega, Madison, WI, USA), following the manufacturer’s instructions. A standard curve was generated using bovine recombinant LDH (Sigma-Aldrich, St. Louis, MO, USA), and LDH levels in the samples were determined by extrapolation from this curve (Fernández-Bravo et al., 2024).

### Analysis of the expression of the genes related to the immune system

Six genes known to be involved in the innate immune response against extracellular pathogens were chosen to assess their transcriptional activity in J774A.1 cells after exposure to *Phialophora* strains, with results compared to non-infected cells. The primers used for gene expression analysis were those described by Murciano et al. (Murciano et al., 2015) and Zhao et al. (Zhao et al., 2017), as listed in **Table 2**. The genes selected encode for cytokines and chemokines (TNF-α and CCL20), apoptosis (TP53), transcription factors (RELA), and pyroptosis-related proteins (NLRP3 and IL-1β). After infecting the J774A.1 cells for 4 h at a MOI of 10, the cells were washed twice with PBS. RNA was then extracted using the GenElute™ Mammalian Total RNA Miniprep Kit (Sigma-Aldrich). cDNA synthesis from the total RNA was performed using the iScript cDNA Synthesis Kit (Bio-Rad Laboratories, Inc., Hercules, CA, USA). Quantitative real-time PCR was carried out on the cDNA with Power SYBR^®^ Green PCR Mastermix (Applied Biosystems^®^, Life Technologies, Glasgow, UK), using a StepOnePlus™ real-time PCR system (Applied Biosystems^®^). Thermal cycling conditions were as follows: initial 5-minute denaturation at 94°C, followed by 40 cycles consisting of 30 seconds at 94°C, 30 seconds at 61°C, 30 seconds at 72°C, and a final extension of 20 seconds at 80°C. The threshold cycle (CT) values were used to calculate the relative RNA expression of the target genes, with glyceraldehyde-3-phosphate dehydrogenase (*GAPDH*) serving as the reference housekeeping gene. Relative gene expression was determined using the delta-delta Ct (2–ΔΔCt) method, based on real-time PCR signal quantification, as previously described (Fernández-Bravo and Figueras, 2022).

**Table 2:** Primers used to target gene expression (Murciano et al., 2015; Fernández-Bravo and Figueras, 2022).

Gene	Sequence (5’-3’)
*GAPDH*	Forward CATGAGAAGTATGACAACAGCCT Reverse AGTCCTTCCACGATACCAAAGT
*TNF-*α	Forward GAGGCCAAGCCCTGGTATG Reverse CGGGCCGATTGATCTCAGC
*RELA*	Forward CCCCAGTCACCTGCTGTTAT Reverse TGGAATCCTGAACCCACTTC
*CCL20*	Forward GCAAGCAACTTTGACTGCT Reverse ATTTGCGCACACAGACAACT
*TP53*	Forward CAGCACATGACGGAGGTTGT Reverse TCATCCAAATACTCCACACGC
*NLRP3*	Forward CGTGAGTCCCATTAAGATGGAGT Reverse CCCGACAGTGGATATAGAACAGA
*IL-1B*	Forward TTCGACACATGGGATAACGAGG Reverse TTTTTGCTGTGAGTCCCGGAG

### Detection of Caspase 1 by Western blotting

Macrophages were infected with *Phialophora* spp. (MOI 10) for 3 h under standard culture conditions. After incubation, cells were washed twice with cold PBS and lysed on ice using RIPA buffer (Thermo Scientific) supplemented with protease inhibitor cocktail (Roche) and 1 mM PMSF. Lysates were incubated on ice for 30 minutes with occasional vortexing, and centrifuged at 14,000 × g for 15 minutes at 4°C. The supernatants were collected, and protein concentration was determined using a BCA protein assay kit (Thermo Scientific, Life Technologies). Equal amounts of total protein (20–30 µg) were mixed with Laemmli sample buffer containing 5% β-mercaptoethanol, boiled at 95°C for 5 minutes, and separated on a 12–15% SDS-PAGE gel. Proteins were transferred onto a PVDF membrane (Millipore, Madrid, Spain) using a wet transfer system at 100 V for 90 minutes at 4 °C.

Membranes were blocked in 5% non-fat dry milk in TBS-T (Tris-buffered saline with 0.1% Tween-20) for 1 h at room temperature, then incubated overnight at 4°C with primary antibodies against cleaved Caspase 1 (Casp 1) p20 (Bio-Rad, Madrid, Spain). After washing, membranes were incubated with HRP-conjugated secondary antibodies (anti-rabbit IgG, 1:3000) for 1 h at room temperature. Band intensities were measured with the ImageJ software program (NIH, MD, USA) (Fernández-Bravo and Figueras, 2022). GAPDH was used as a loading control in the Western blot.

### Biofilm formation

To investigate biofilm formation following macrophage infection, J774A.1 macrophage cells were seeded in 24-well plates at a density of 1x10^5^ cells per well, and incubated overnight at 37°C with 5% CO_2_ to allow cell attachment. The following day, the cells were infected with the *Phialophora* strains at MOI 10 for 1 h. After the incubation period, macrophages were washed twice with PBS to remove unbound conidia. Fresh culture medium was then added, and the cells were incubated for an additional 24 h to promote biofilm development.

To assess biofilm formation, the cells were washed gently with PBS to eliminate non-adherent conidia. Biofilms were stained with a 0.1% Gentian violet solution for 20─30 min at room temperature. After staining, excess dye was removed by washing with PBS. The attached biofilm was then solubilized by adding 95% ethanol to each well and incubating for 30 min at room temperature to release the Gentian violet. The optical density (OD) of the solubilized biofilm was measured at 570 nm using a microplate reader to quantify the biofilm biomass (Soussa et al., 2022).

### Antifungal susceptibility pattern

The *in vitro* susceptibility of *Phialophora* spp. was performed using broth microdilution method according to the reference Clinical and Laboratory Standards Institute (CLSI) guidelines, document M38 3rd edition (Clinical and Laboratory Standards Institute (CLSI), 2017). *Aspergillus flavus* ATCC204304 was included as a quality control strain. Minimum inhibitory concentrations (MICs) were determined for fluconazole (FLZ, Sigma-Aldrich, St. Louis, MO, EEUU), itraconazole (ITZ, St. Louis, MO, EEUU), posaconazole (PSZ, Sigma-Aldrich, St. Louis, MO, EEUU), voriconazole (VRZ, Sigma-Aldrich, St. Louis, MO, EEUU), amphotericin B (AMB, Sigma-Aldrich, St. Louis, MO, EEUU), 5-fluorocytosine (5-FC, Sigma-Aldrich, St. Louis, MO, EEUU), olorofim (OLF, F2G, Manchester, UK), and terbinafine (TRB, Sigma-Aldrich, St. Louis, MO, EEUU). For the echinocandins, anidulafungin (AND, Pfizer Inc., Madrid, Spain), caspofungin (CSP, Merk & Co., Inc., Rahway, EEUU), and micafungin (MCF, Sigma-Aldrich, St. Louis, MO, EEUU) minimal effective concentrations (MECs) were determined. MICs and MECs were read after 8 days of incubation at 30°C, considering the maximum temperature for growth of *P. submersa* (Torres-Garcia et al., 2023).

### Stress sensitivity assay

We assessed the cellular stress sensitivity of five *P. submersa* strains (**Table 1**). To do so, 10 μl of conidial suspensions (5x10^5^ conidia/mL) were cultured by inoculating a single drop onto supplemented potato dextrose agar (PDA) plates and incubated for two weeks. Osmotic stress was tested by supplementing PDA with sorbitol (2M and 3M), potassium chloride (0,5M and 1M), or sodium chloride (0,5M and 1M). Cell wall-destabilizing stress was induced using calcofluor white (CFW; 200 and 250 μg/mL) and Congo Red (CR; 1 and 1,25 mg/mL). Membrane stress conditions were done with sodium dodecyl sulphate (SDS; 50 and 100 μg/mL). Sensitivity to the different stresses was evaluated by measuring radial growth after two weeks of incubation at 25°C using the software ImageJ (version 1.54g).

### Statistical analysis

All experiments were replicated at least 3 times. Statistical analyses were performed by using *t-*test and 1-way ANOVA with Tukey *post hoc* test, or by 2-way ANOVA with Sidak *post hoc* test with GraphPad Prism 6.0 (GraphPad Software, CA, USA). *P* value of <0.05 was considered statistically significant.

## Results

### Phagocytosis and intracellular survival after macrophage infections with the fungal strains

**Figure 1:**
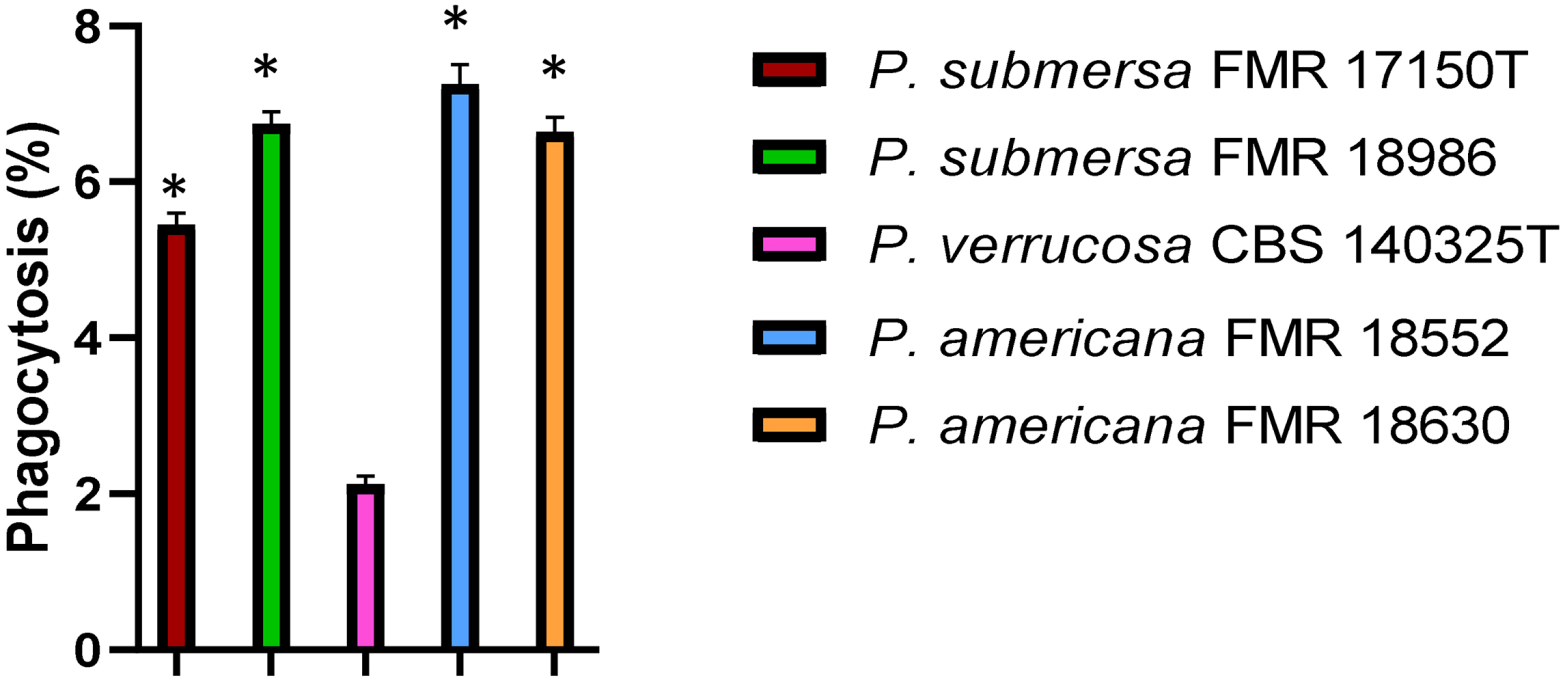
Phagocytosis assay after infection with the *Phialophora* strains at 1 h post-infection at MOI 10. Data were analyzed by using the Student’s two-tailed t-test and by 1- way ANOVA with Tukey *post hoc*. Asterisks indicate statistically a significant difference P < 0.05*.

The phagocytic activity of J774A.1 macrophages was significantly influenced by the *Phialophora* species involved in the infection. Upon infection with *P. verrucosa*, the percentage of phagocytosis was notably lower compared to the other two species tested (p > 0.05). In contrast, infection with *P. americana* led to the highest levels of phagocytosis, with macrophages efficiently engulfing the fungal cells. The phagocytic activity observed in macrophages infected with *P. submersa* was intermediate, with phagocytosis levels falling between those of *P. verrucosa* and *P. americana* (**Figure 1**).

**Figure 2:**
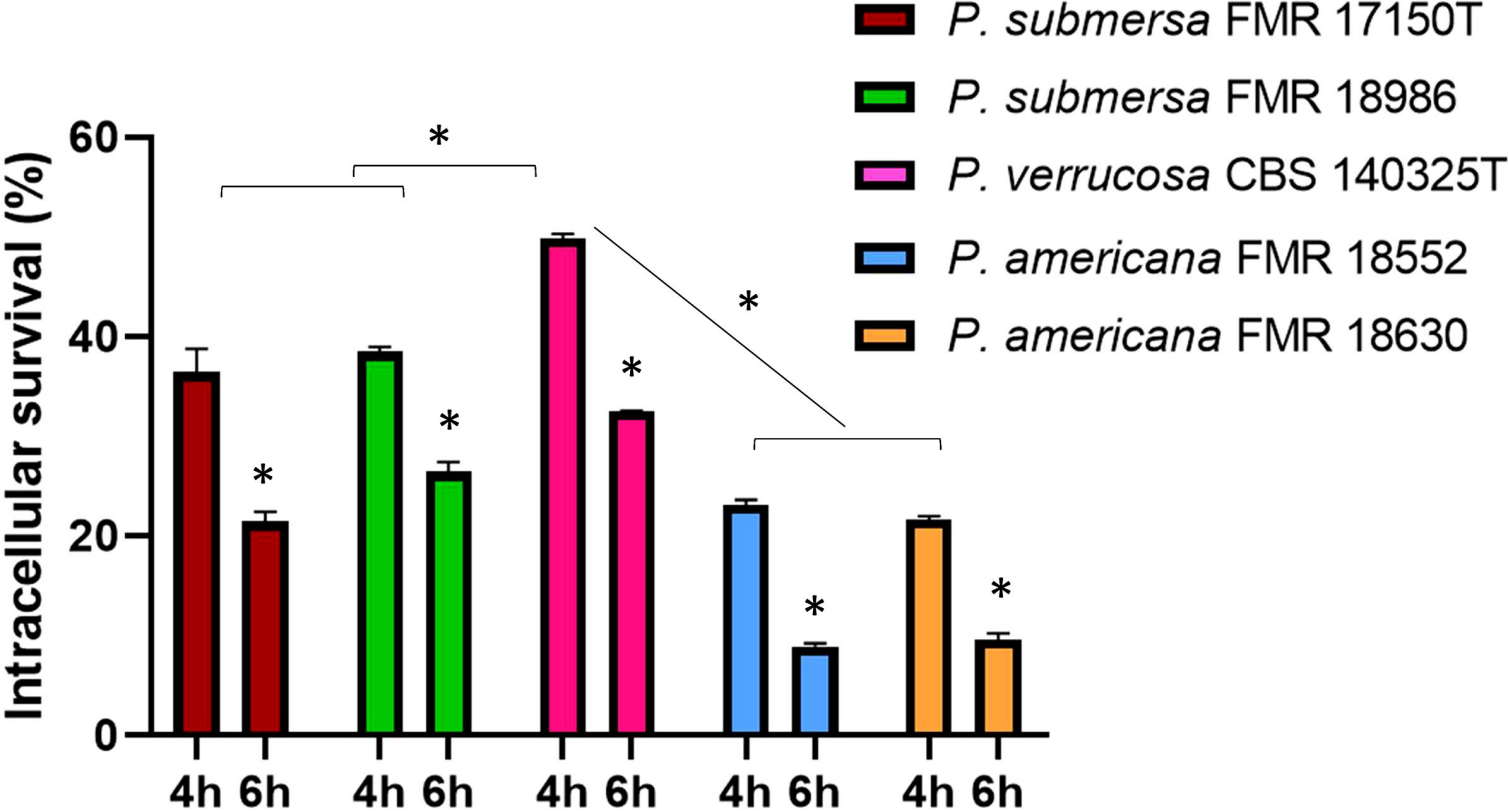
Intracellular survival after infection with the *Phialophora* strains at 4 and 6 h post-infection at MOI 10. Data were analyzed by using the 2- way ANOVA with Sidak *post hoc* test. Asterisks indicate a statistically significant difference P < 0.05*.

Intracellular survival was assessed at 4 and 6 h post-infection, revealing that, regardless of the infecting *Phialophora* species, survival was higher at 4 h and decreased significantly by 6 h (p < 0.05). Among the species tested, *P. verrucosa* showed the highest intracellular survival, followed by *P. submersa*, with *P. americana*, irrespective of the strain used (p < 0.05) (**Figure 2**).

### Cell damage after macrophage infection at different times

**Figure 3:**
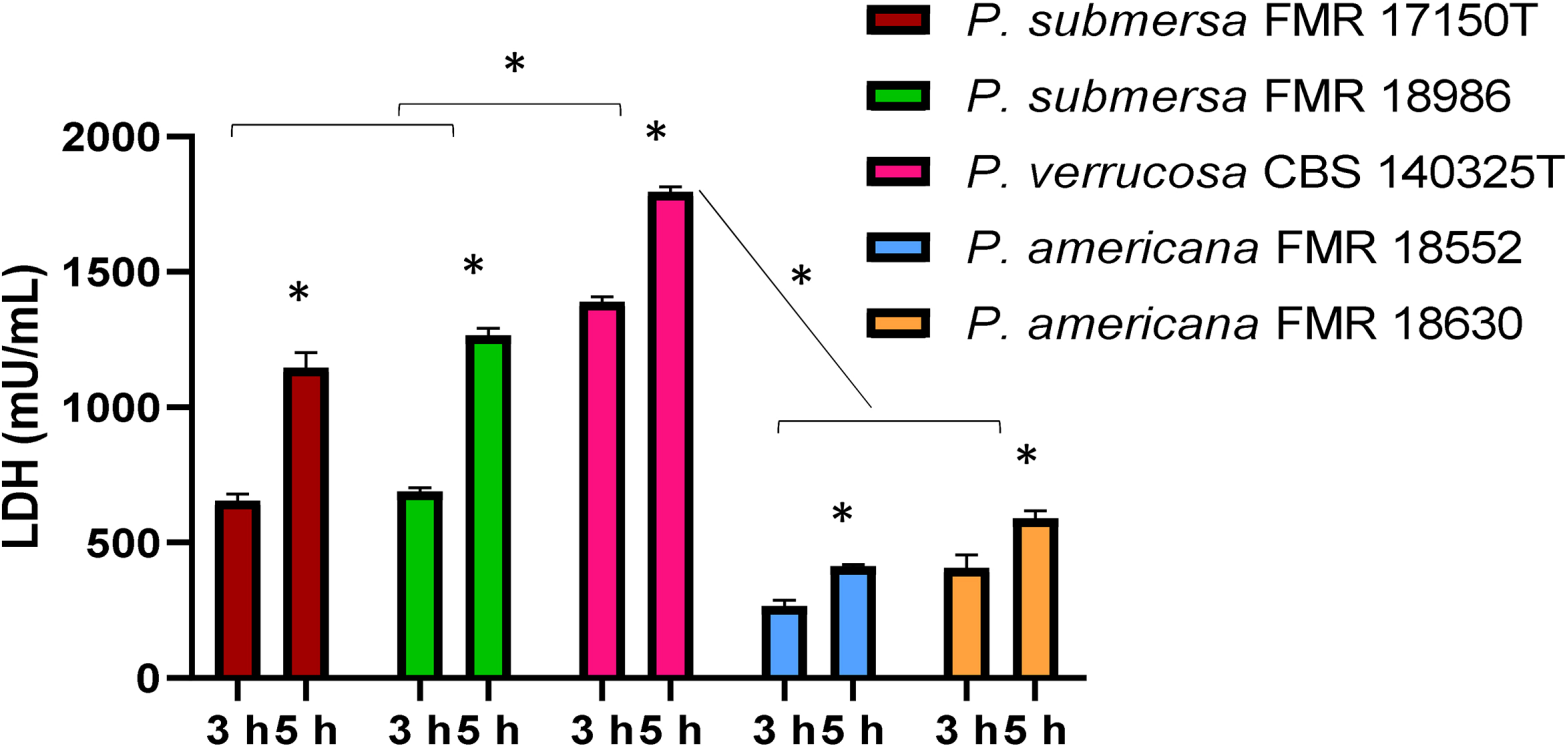
Detected J774.1A cell damage induced at 3 and 5 h by the *Phialophora* infections at MOI 10 in cells, measured by the release of lactate dehydrogenase (LDH) enzyme. Data were analyzed by using the 2- way ANOVA with Sidak *post hoc* test. Asterisks indicate a statistically significant difference P < 0.05*.

Cellular damage, measured by LDH enzyme release, was lower at 3 h post-infection but increased significantly by 5 h (p < 0.05), regardless of the *Phialophora* species infecting the macrophages. Among the species tested, the highest level of cellular damage was observed following infection with *P. verrucosa*, followed by *P. submersa*, and finally *P. americana*, irrespective of the strain used (**Figure 3**).

### Macrophage cell line (J774.1A) gene-expression after infection with the fungal strains

#### Genes for cytokines and chemokines

**Figure 4:**
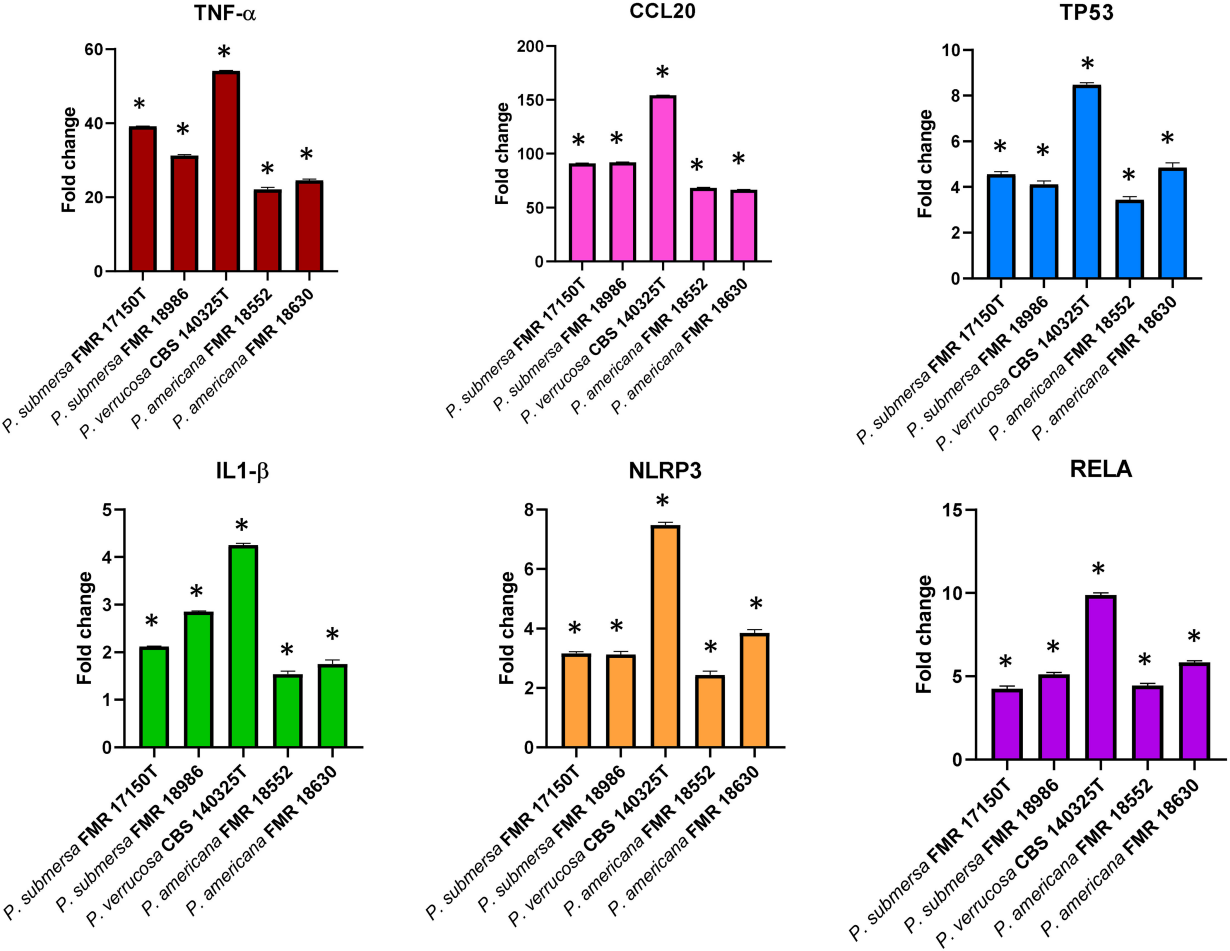
Gene expression profile of J7741. A cells in relation to the non-infected cells induced by *Phialophora* infections at MOI 10 determined by RT-qPCR. Transcript levels of the genes were normalized to the expression of the *GAPDH* gene. Expression fold change with respect to the non-infected cells was calculated using the comparative ΔΔCt method. Data were analyzed by using the Student’s two-tailed t-test and by 2- way ANOVA. Asterisks indicate a statistically significant difference P < 0.05*.

All infections resulted in the expression of genes encoding the cytokine TNF-α, and the chemokine CCL20 in J774A.1 macrophages, with significant differences (p < 0.05) in transcription patterns compared to non-infected cells (**Figure 4**). Furthermore, the expression of *TNF-α* and *CCL20* were notably higher following infection with *P. verrucosa* compared to the strains of *P. submersa* and *P. americana* (p < 0.05). Additionally, the expression levels of both genes were significantly higher after infection with *P. submersa* compared to *P. americana.* No significant differences were found between strains of the same species (**Figure 4**).

#### Gene involved in apoptosis

The transcriptional expression of the *TP53* apoptosis gene was significantly elevated after infection with all three species in comparison with the non-infected cells. *P. verrucosa* caused a greater overexpression of *TP53* compared with *P. submersa* and *P. americana* (p <0.05) (**Figure 2**). No significant differences in *TP53* expression were observed between infections with *P. americana* and *P. submersa*, independently of the strain used (**Figure 4**).

#### Genes related to the inflammasome and pyroptosis

The *NLRP3* and *IL-1β* genes, which are involved in pyroptosis, a type of cell death mediated by inflammasome activation, were significantly upregulated in J774A.1 cells following infections with all strains compared to non-infected controls (p < 0.05). The expression levels of *NLPR3* were significantly highest after *P. verrucosa* infection, followed by *P. submersa* and *P. americana*. The expression levels of *IL-1β* were also higher after *P. verrucosa* infection, but were similar after infection with *P. americana* and *P. submersa* (**Figure 4**).

#### Genes related to the transcription factor RELA

All infections resulted in higher expression levels of the *RELA* gene, which is related to cytokine production, in comparison with non-infected cells (p < 0.05). However, *P. verrucosa* infection led to the highest overexpression of the *RELA* gene, followed by *P. submersa* and *P. americana.* Regardless of the strains, these levels were higher after infection with *P. submersa* than *P. americana* (**Figure 4**).

### Detection of Casp 1 after *Phialophora* infection

**Figure 5:**
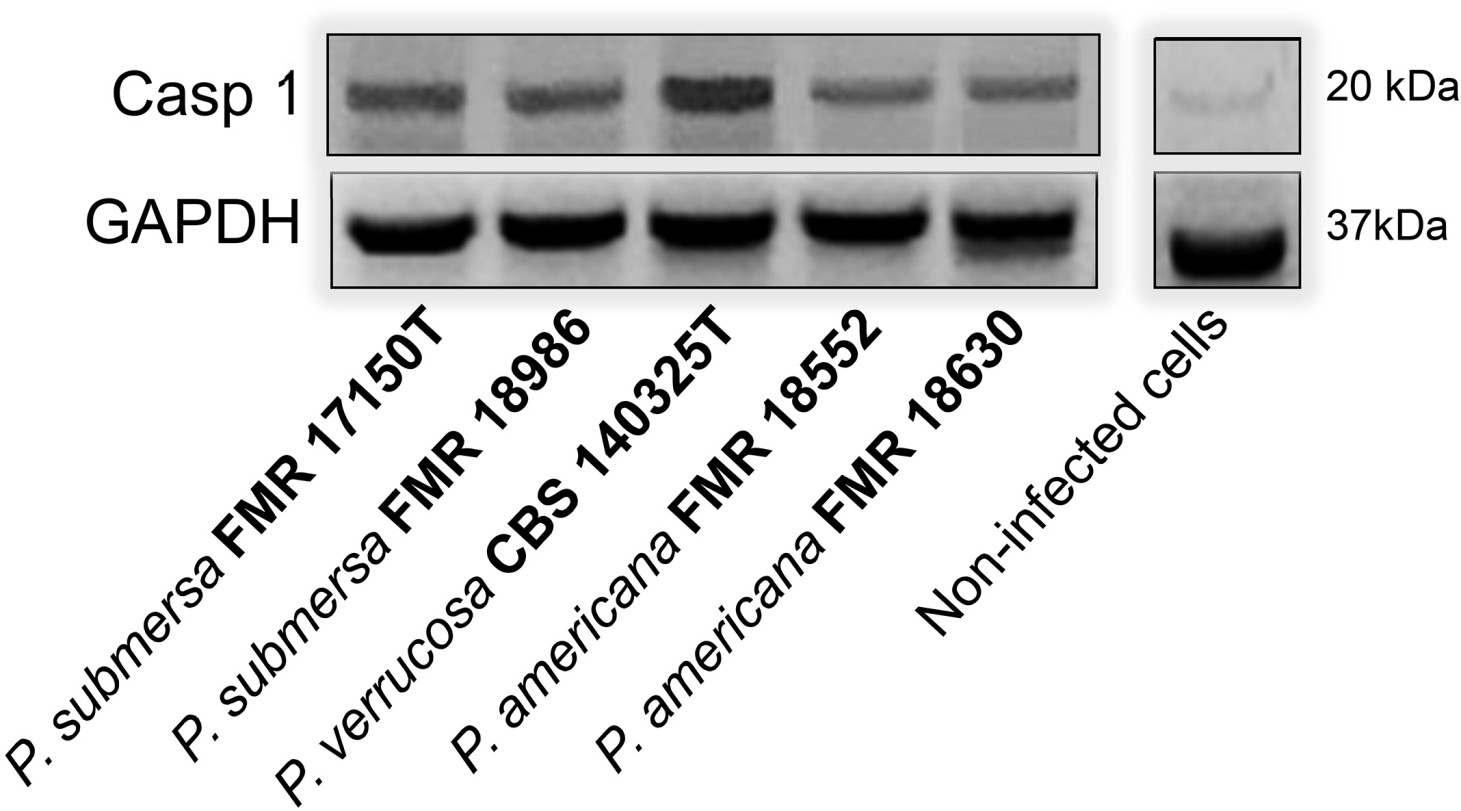
Activation of Caspase 1 by *Phialophora* strains in macrophages measured by Western blotting.

The activation of Casp 1 were analyzed by Western blotting using an antibody that recognized the protein. The results demonstrated that, in macrophages J7741.A, all strains induced de activation of Casp 1 at 3 h postinfection. However, the band intensity was higher after infection with *P. verrucosa* (**Figure 5**).

### Biofilm formation assay with gentian violet staining after macrophage infection

**Figure 6:**
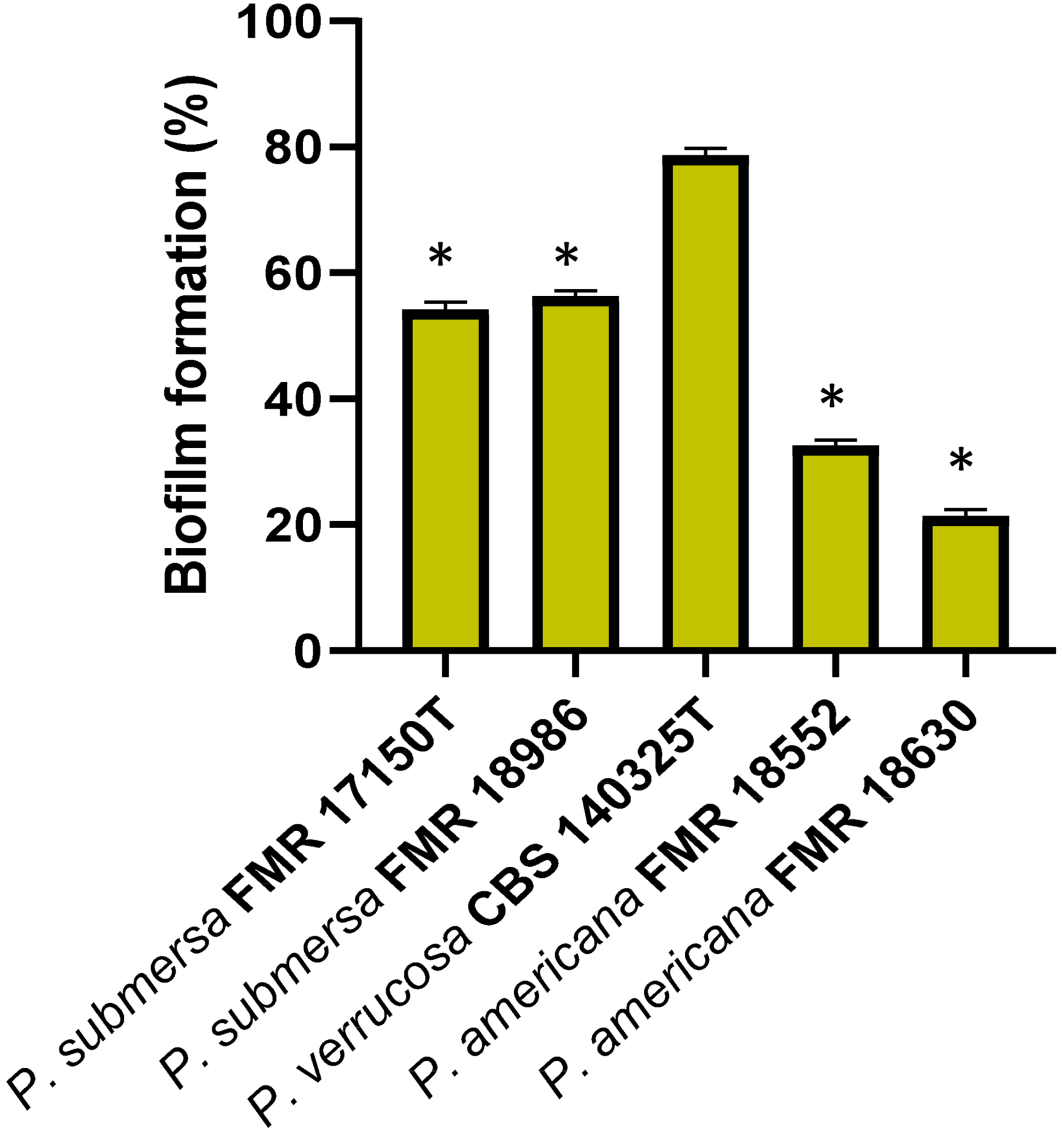
Percentage of biofilm formation after 24 h post-infection with the *Phialophora* strains. Data were analyzed by using the Student’s two-tailed t-test and by 1- way ANOVA with Tukey *post hoc*. Asterisks indicate statistically significant difference P < 0.05*.

Biofilm formation by *Phialophora* strains on previously adherent macrophage cultures was assessed by Gentian violet staining. The results showed that the *P. verrucosa* strain produced significantly more biofilm (p > 0.05) compared to the other strains tested, while *P. submersa* strains exhibited a higher biofilm production than those of *P. americana* (**Figure 6**). This test demonstrated that *Phialophora* species have a notable capacity for biofilm formation, with production levels varying among species, and *P. americana* displaying the lowest biofilm-forming ability.

### Antifungal susceptibility

The antifungal susceptibility results for the three *Phialophora* species (**Table 3**) showed that TBF exhibited the highest antifungal activity against all strains tested, with MIC values ranging from 0.12 µg/mL to 0.5 µg/mL. The lowest MIC value was observed against *P. verrucosa* (MIC = 0.12 µg/mL). All azoles demonstrated potent antifungal activity (MIC ≤ 0.5 µg/mL) across the species tested, with the exception of FLZ, which showed poor activity (MIC ≥ 64 µg/mL), and a single *P. americana* strain (FMR18630) that exhibited a MIC of 1 µg/ml for VRZ. The modal MIC of AMB was 2 µg/ml against *P. submersa*, while *P. americana* and *P. verrucosa* showed slightly higher resistance, with a MIC of 4 µg/mL. Susceptibility to OLF and 5-FC was also low in all *Phialophora* strains tested, with MIC values ranging from 1 to ≥16 µg/mL. Echinocandins showed strain-dependent MECs, with values ranging from 0.06 to 4 µg/mL across all species, except for *P. verrucosa*, which was resistant to the three tested echinocandins (MEC ≥ 16 µg/mL). Notably, MCF was the only echinocandin that demonstrated antifungal activity against the *P. americana* and *P. submersa* strains tested, with MIC values between 0.06 and 0.5 µg/mL.

**Table 3:** *In vitro* antifungal susceptibility profiles of the *Phialophora* species tested.

MIC and MEC (μg/ml)											
Species (strain)	PSZ	ITZ	VRZ	FLZ	AMB	5-FC	TBF	OLF	MCF	AND	CSP
*P. americana* (FMR 18552)	0.25	0.5	0.5	64	4	8	0.5	>16	0.12	1	0.5
*P. americana* (FMR 18630)	0.25	0.25	1	64	4	16	0.25	>16	0.06	0.12	1
*P. submersa* (FMR 17150)	0.25	0.5	0.5	>64	2	2	0.25	2	0.5	1	0.5
*P. submersa* (FMR 18986)	0.25	0.5	0.5	>64	2	1	0.25	2	0.5	2	4
*P. verrucosa* (CBS 140325)	0.25	0.5	0.5	64	4	>16	0.12	2	>16	>16	>16
*A. flavus* (ATCC 204304)	0.25	0.5	0.5	64	0.5	2	0.06	>0.03	>16	4	>16

AMB, amphotericin B; AND, anidulafungin; CSP, caspofungin; FLZ, fluconazole; 5-FC, 5-fluorocytosine; ITZ, itraconazole; MCF, micafungin; PSZ, posaconazole; TRB, terbinafine; OLF, olorofim; VRZ, voriconazole.

### Stress response and adaptation of *P. submersa*

**Figure 7:**
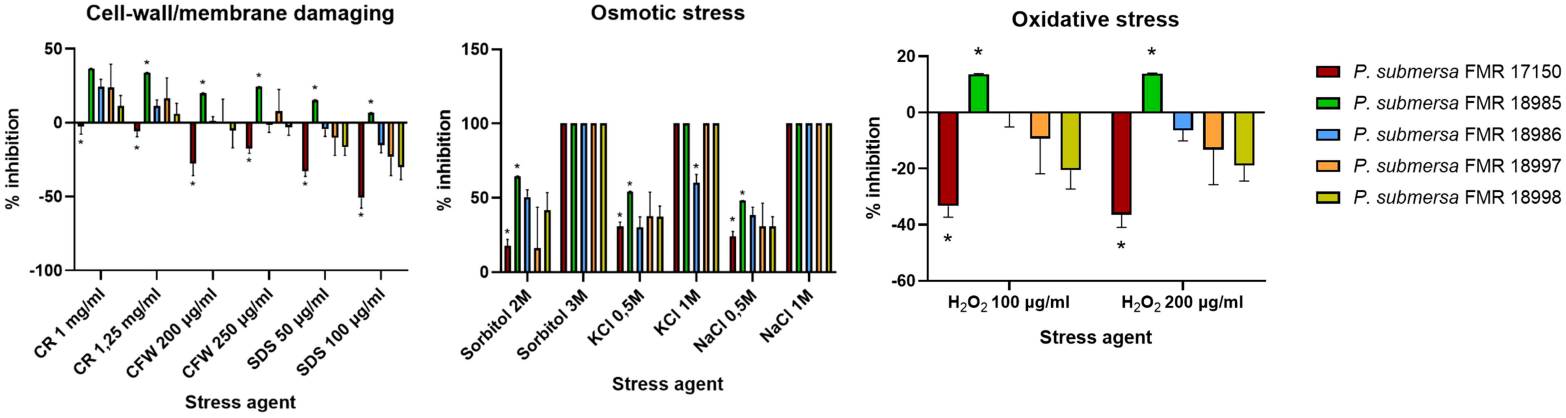
Rate inhibition of growth in *Phialophora submersa* under different stress conditions. Data were analyzed using 2-way ANOVA. Asterisks indicate a statistically significant difference P < 0.05*.

Among the five tested strains of *P. submersa*, FMR 17150 exhibited the highest resistance to a range of stress conditions, except for cationic and salt stress. None of the strains showed growth on PDA supplemented with 3M sorbitol or 1M NaCl, indicating complete growth inhibition under these conditions (100% inhibition). In contrast, growth on PDA supplemented with 1M KCl was limited, with only FMR 18986 capable of growing under this condition. Under cell wall-destabilizing stress conditions (CFW and CR), FMR 17150 showed notable resistance, displaying even enhanced growth compared to control conditions. However, the other strains were sensitive to CR, showing reduced growth. All strains demonstrated resistance to membrane-destabilizing stress (SDS) and oxidative stress (H_2_O_2_) conditions, suggesting a general resilience of *P. submersa* to these environmental challenges (**Figure 7**).

## Discussion

The findings of this study provide new insights into the pathogenic potential and host-pathogen interactions of *Phialophora* species, with a particular focus on *P. americana, P. submersa*, and *P. verrucosa*. These species exhibited varying abilities to infect macrophages, form biofilms, and induce cellular damage, highlighting their different virulence profiles. Additionally, we observed changes in the transcription levels of key immune system genes, which may play a role in the host’s response to infection and further elucidate the pathogenesis of these species.

Phagocytosis assays revealed that macrophages internalized *P. verrucosa* significantly less efficiently than *P. americana*, with *P. submersa* showing intermediate uptake, irrespective of the strains tested. This supports previous findings indicating that fungal species differ in their ability to evade innate immune responses, with some fungi expressing surface proteins that reduce recognition by immune cells (Gordon and Taylor, 2005; Mira et al., 2017). The reduced phagocytosis level observed with *P. verrucosa* may be attributed to its ability to inhibit immune cell recognition through the modulation of host signaling pathways or the expression of surface proteins that evade immune detection (Gordon and Taylor, 2005). In contrast, the higher phagocytic uptake of *P. americana* implies a less effective evasion strategy, correlating with its lower intracellular survival.

In terms of intracellular survival, *P. verrucosa* demonstrated the highest survival at 4 h post-infection, followed by *P. submersa* and *P. americana*. This pattern aligns with the hypothesis that more virulent species can better withstand phagolysosomal conditions through mechanisms such as inhibition of phagosomal acidification or antioxidant defense systems (Borges et al., 2018).

In addition to the observed differences in phagocytosis and survival, transcriptional analysis of key immune-related genes also revealed significant variations in the macrophage response to these *Phialophora* species. The cytokines and chemokines TNF-α and CCL20, which play critical roles in inflammation and immune cell recruitment, were unregulated following infection with all *Phialophora* species, demonstrating that these pathogens elicit an inflammatory response from the host immune system. Notably, *P. verrucosa* induced the highest levels of *TNF-α* and *CCL20* expression, suggesting a more robust inflammatory response. These cytokines are known to be involved in the recruitment of additional immune cells, potentially contributing to the persistence of infection (Cugini et al., 2021). On the other hand, *P. americana* and *P. submersa* induced lower levels of these cytokines, which could reflect differences in their ability to modulate host immune responses or evade immune detection. The upregulation of the apoptosis-related gene *TP53* and the transcription factor gene *RELA* in response to all *Phialophora* species further emphasizes the immune response activation, as TP53 and RELA are key regulators of cell death pathways that can influence pathogen survival within the host (Maderna and Godson, 2003). RELA/p65 plays a central role in the regulation of inflammatory and immune responses. Upon activation of the NF-κB pathway, RELA translocates to the nucleus, where it drives the transcription of various pro-inflammatory genes, including *TNF-α* or *IL-1β* and *CCL20*, a chemokine involved in the recruitment of CCR6+ immune cells. The promoter region of *CCL20* contains NF-κB binding motifs, and its expression is known to be directly regulated by RELA (Maderna and Godson, 2003; Borges et al., 2018; Cugini et al., 2021).

The pyroptosis-related genes *NLRP3* and *IL-1β* were also significantly upregulated in response to infection, indicating the activation of inflammasomes and the subsequent release of pro-inflammatory cytokines that are crucial for the host’s defense against infection (Borges et al., 2018). The highest expression of *IL-1β* was observed following the infection of *P. verrucosa*, followed by *P. submersa*, which may suggest a heightened inflammatory response when these species are present in the infection. To further investigate this pathway, we performed a Western blot analysis to assess Casp 1 activation. While only minor differences were observed among most species, *P. verrucose* notably induced higher levels of cleaved Casp 1, aligning with its strong *IL-1β* expression. These results support the idea that inflammasome activation, particularly via Casp 1, may be species-dependent and more prominent during *P. verrucosa* infection (Borges et al., 2018).

Interestingly, recent comparative genomic analyses by Song et al (Song et al., 2021). identified the presence of multiple genes in *Phialophora* species potentially involved in immune evasion and host-pathogen interactions. Among these, CARD9-related pathways were highlighted as particularly relevant, given the central role of CARD9. CARD9 acts as a key adaptor protein in the signaling cascade triggered by C-type lectin receptors, leading to NF-κB activation and pro-inflammatory cytokine production. Mutations or deficiencies in CARD9 are known to predispose individuals to deep and disseminated fungal infections (Sobianski-Herman et al., 2024). The conservation of CARD9-interacting domains in virulent *Phialophora* strains, including *P. verrucosa* and *P. americana*, suggests that these fungi may exploit or modulate CARD9-mediated pathways to influence host immune responses. This could partially explain the differential expression of inflammatory markers observed in our macrophage infection assays and further supports the hypothesis that *Phialophora* species have evolved distinct mechanisms to interact with innate immunity depending on their ecological origin and pathogenic potential. Further research is necessary to confirm this issue.

Biofilm formation is a critical virulence factor correlated with the pathogenicity of many fungi, as it protects from host immune responses and antimicrobial treatments (Cugini et al., 2021). In our study, *P. verrucosa* produced the highest biofilm biomass, with *P. americana* forming the least amount of biofilm. This is consistent with the notion that biofilm formation contributes to fungal virulence by protecting cells from host defenses, including macrophages (Nobile and Johnson, 2015). Strong biofilm formation in *P. verrucosa* may partly explain its higher intracellular survival, as biofilm cells are known to exhibit increased resistance to both phagocytosis and antimicrobial agents (Finkel and Mitchell, 2011). The intermediate biofilm formation observed with *P. submersa* suggests that the ability to form biofilms may vary among *Phialophora* species, which could influence their virulence, survival in host tissues, and clinical outcomes in case of infection.

Cellular damage, assessed via LDH release, increased over time for all species, with *P. verrucosa* inducing the greatest damage, followed by *P. submersa* and *P. americana.* This test is indicative of host cell membrane disruption, often a consequence of the inflammatory response triggered by fungal infection (Maderna and Godson, 2003). This aligns with other virulence metrics, emphasizing the link between inflammation, biofilm-associated protection, and host cell disruption. The higher levels of cellular damage induced by *P. verrucosa* may reflect its more aggressive interaction with host cells, potentially involving the secretion of virulence factors that disrupt cell membranes or induce necrosis. In contrast, *P. americana* caused less cellular damage, possibly due to its less aggressive interaction with macrophages or a less pronounced inflammatory response. The intermediate cellular damage induced by *P. submersa* further supports the notion that this species exhibits virulence traits that are more similar to *P. americana* than to *P. verrucosa*.

Stress response and adaptation to a changing environment are critical for the survival of fungal species in dynamic natural environments and host niches (Brown et al., 2014; Jacobsen et al., 2018). Exposure of microorganisms to specific incremental stress enhances their resistance to that stress factor and other stresses (Gasch, 2003). Our results show that stress conditions involving the cell wall and membrane have a bigger impact on growth than the other challenging conditions. CR, a β-1,3-glucan-binding agent, and CFW, a chitin-binding agent, directly counteract the cell wall stress response itself. Different studies have shown that strains with lowered chitin levels in their walls seem more resistant to CR and CFW (Ram and Klis, 2006), being strain-dependent, as our results show. Interestingly, only the strain FMR17150 of *P. submersa* showed higher mycelial growth in the presence of CR and CFW.

Antioxidant defense is crucial in maintaining redox homeostasis and consist in several enzymatic activities, such are SODs, catalases, and thioredoxin/glutathione systems, among others, as well as non-enzymatic molecules such melanin. The antioxidant armamentarium in fungi is directly linked to fungal pathogenesis by attenuating host cellular ROS activity (Yaakoub et al., 2022). Overall, *P. submersa*, which is a more virulent species than *P. americana*, has shown, in general, a high resistance to cellular stresses. Resistance to SDS, a common detergent that permeates cell membranes and activates stress responses (Schroeder and Ikui, 2019), as seem for oxidative stress and CFW tests. However, results must be interpreted in a strain-dependent way and not as species characteristic since *P. submersa* FMR 17150 showed the most resistance to SDS, H_2_O_2_, CR and CFW, correlating with its strong biofilm formation. These traits likely enhance intracellular persistence (Matsumoto et al., 2023).

Finely, our antifungal susceptibility testing did not reveal clear species-specific patterns, although *P. verrucosa* was notably less susceptible to echinocandins. This may be linked to the clinical origin of the *P. verrucosa* strain used in our study, in contrast to the environmental origin of the other strains. Supporting this, Li et al. (Li et al., 2014) reported that clinical strains identified as *P. verrucosa* exhibited higher resistance than those environmental strains. Nonetheless, this data should be approached with caution, because clinical or environmental strains identified as *P. verrucosa* have later been reclassified mainly as *P. americana* and *P. chinensi*s (Li et al., 2017; De Hoog et al., 2020; Ahmed et al., 2021). Recent literature suggests that *P. verrucosa* has yet to be reliably isolated from environmental sources, like its counterparts *P. expanda* and *P. tarda* (Li et al., 2017; De Hoog et al., 2020; Ahmed et al., 2021; Song et al., 2021).

## Conclusions

This study focuses on the pathogenicity of *P. submersa* compared to *P. verrucosa* and *P. americana* on macrophage infections. By integrating macrophage infection assays, gene expression analyses, stress response evaluation, and antifungal susceptibility testing, we delineate distinct virulence profiles and stress tolerance mechanisms that could reflect their ecological origins and pathogenic capabilities. *Phialophora verrucosa* exhibited the highest virulence, with strong biofilm formation, extensive cellular damage, and superior intracellular survival. In contrast, *P. americana* showed the lowest virulence, with minimal biofilm production, lower cellular damage, and reduced intracellular macrophage survival. *Phialophora submersa* displayed an intermediate virulence profile, with moderate biofilm production and intracellular persistence. The immune response to these black yeasts also differed across species, with variations in the expression of cytokines, chemokines, apoptosis-related genes, and pyroptosis markers, further reflecting their differing virulence profiles. This suggests that the immune response is closely tied to the species-specific pathogenicity of *Phialophora*. All these findings suggest that, despite its environmental origin and its limitation to grow at human body temperature, *P. submersa* presents pathogenic potential to cause infection. These findings support the hypothesis of Song et al (Song et al., 2021), which suggests that all *Phialophora* strains, either clinical or environmental, possess a comparable potential to cause infection in humans. Understanding all these factors, particularly addressed to the stress resistance observed in *P. submersa* strains, may help inform the development of targeted therapeutic strategies and provide insights into the role of these fungi in infections.

Variations in stress response and resistance patterns observed in *P. submersa* may be attributed to the genetic variability detected among strains of this species, as well as its ability to adapt to the environmental conditions of its native habitats (**Table 1**; Torres-Garcia et al., 2023). Further genomic investigations could help to clarify and confirm this correlation.

## Data Availability

The original contributions presented in the study are included in the article, further inquiries can be directed to the corresponding author. Data regarding fungal strains presented in the study are deposited in the NCBI GenBank repository, accession numbers: ON009864; ON009944; ON009866; ON009946; ON009860; ON009940; ON009861; ON009941; KF881960; KF971761.
